# Health problems and help-seeking activities of methadone maintenance clients at Auckland Methadone Service (AMS): potential for community pharmacy service expansion?

**DOI:** 10.1186/1477-7517-2-25

**Published:** 2005-11-13

**Authors:** Janie Sheridan, Amanda Wheeler, Carina Walters

**Affiliations:** 1 The School of Pharmacy, University of Auckland, Private Bag 92019, Auckland, New Zealand; 2 Clinical Research Resource Centre, Waitemata District Health Board, Auckland, New Zealand; 3 Auckland Methadone Service, Waitemata District Health Board, Auckland, New Zealand

## Abstract

**Background:**

In general the health of methadone clients has been found to be poorer than that of the general population. In New Zealand specialist drug services are not funded to provide primary healthcare services. Many health conditions could potentially be managed by community pharmacists who have frequent contact with this client group. This study sought to explore the health problems suffered by methadone clients, who they sought help from, and the potential for greater involvement of pharmacists.

**Methods:**

Self-completion questionnaire of methadone maintenance clients managed in specialist care in Auckland, New Zealand.

**Results:**

The most common health problem experienced by these clients in the past three months was sweating (70.0%), and more than half of the respondents also reported experiencing headache, fatigue and depression. The least frequently experienced conditions were hay fever (12.9%) and abscesses (12.1%). Respondents indicated that the top three choices from whom they would seek help were GP (56.7%), the client's partner (31.6%) and community pharmacists (27.9%). Barriers to seeking help from pharmacists included issues around cost, perceptions of pharmacist knowledge and skills, privacy and confidentiality.

**Conclusion:**

Methadone clients in this study indicated that they suffered a number of general health problems, and in many cases were likely to seek help from a GP or their own partner, before seeking help from pharmacists. However, for over one quarter of respondents the pharmacist was in the top three from whom they would seek advice. Any barriers towards consulting pharmacists, in the main seem to be resolvable.

## Background

In general the health of drug users has been found to be poorer than that of the general population. Ryan and White [1] noted that the health status of clients entering a public methadone programme in Australia was generally poorer on measures of physical and psychological health than that of the general population. A New Zealand study of self-assessed health status (using SF36) found this was significantly worse in methadone patients than the New Zealand population mean score, and 75% of the sample had engaged in help-seeking activities around general health issues for themselves [2]. With regard to psychological health, Brienza et al found that 42% of methadone patients met criteria for major depression [3].

Research focusing on more specific aspects of methadone client health has pointed to significant poor health and morbidity. In a study of nutrition, methadone clients were found to prefer sweet foods and to consume in excess of the daily recommended proportion of mono and disaccharides [4]. A similar finding was noted in Australia where women in methadone treatment had a high sugar and low fibre intake [5], and Best and colleagues noted that among methadone patients in their study, 3% reported no "eating events" in the previous three days and 27% had eaten no cooked meals [6]. Other health problems which particularly affect methadone clients include sweating and constipation [7], sleep disturbances [8,9] and dental problems [10-12]. Lack of treatment for such problems may result in increased health-related harm to drug users.

In New Zealand, community pharmacists have long been involved in the provision of harm reduction services to drug misusers – either through their role in the provision of needle exchange [13,14] or through the dispensing of methadone prescriptions for the management of opiate dependence. In 2003 it was estimated that around 1 in 5 community pharmacies provided a needle exchange service (personal communication – Charles Henderson) and approximately a half of community pharmacists work in pharmacies which dispensed methadone prescriptions (personal communication – Linda Bryant). These figures are similar to those found in England and Wales [15] and represent a major contribution of the profession to providing support and healthcare to drug users.

The provision of these services brings community pharmacists into frequent contact with injecting drug users and opiate dependent patients [16], thus creating potential opportunities for further intervention around a wide range of issues. For example, many needle exchange pharmacies provide written and verbal information on testing for blood borne viruses, accessing treatment, avoiding overdose and safer injecting [17]. Such interventions are obvious in relation to the client group. However, it may be argued that community pharmacists having successfully taken on such roles, have tended to focus on the drug user in terms of their drug use and route of use, and have possibly paid less attention to the general health issues of drug users. Such health problems may be the same kind of health problems suffered by the general population (e.g. coughs, colds, headache etc) or may be directly related to their drug use. There is however, the issue of stigmatisation of drug users, in which legitimate requests for over-the-counter remedies for problems such as pain, coughs and colds may be treated with suspicion, as many of these products contain substances liable to abuse [18,19].

This present study has sought to explore some of these issues in a group of methadone clients at Auckland Methadone Service (AMS), New Zealand. AMS provides a methadone maintenance, methadone reduction and methadone detoxification service to opiate dependent clients in a catchment area of almost 1.2 million [20-22]. At the time of conducting the study (Mid January to mid April 2005), AMS was funded to provide services for 989 methadone clients. Around three quarters are managed within the service having their methadone prescribed to them by AMS medical officers and seeing a case manager at least once every three months at a Community Alcohol and Drug service (CADS) clinic of their choice, the remaining quarter managed in primary care having their methadone prescribed by authorised general practitioners and routinely accessing AMS for further consultation. GP managed clients have been stabilised within the service and are considered to be more stable with regard to drug use and behaviour than those managed within AMS.

AMS is not funded to provide general healthcare to clients, so those managed within the service who do not have a GP to consult about their health may need to use other health professionals or lay people for help with such problems. In addition, in New Zealand, GP care incurs a cost to the patient at the point of access and may be seen as a barrier to accessing treatment; treatment through AMS is free of charge.

Another health professional trained to provide advice, treatment and referral is the community pharmacist. Consulting the pharmacist is free of charge and no appointment is needed. However, if medicines are required they must either be purchased from the pharmacy or obtained on prescription, both of which will incur a cost. In theory, with methadone clients seeing pharmacists several times a week to collect their prescribed methadone there are numerous opportunities for clients to consult pharmacists about their health, or for pharmacists to proactively enquire about health issues known to be of particular problem for methadone clients.

There are a number of potential barriers to methadone clients seeking healthcare from community pharmacists. Research into drug users' views of accessing methadone and needle exchange services through pharmacies have described several barriers. These include the attitudes of the pharmacist and staff and lack of privacy [23-25]. In addition, many pharmacists report not wishing to provide these services [15,16] and thus maybe unlikely to be willing to provide primary healthcare services to drug users.

In New Zealand, no data exist about the incidence of general health problems suffered by methadone patients, nor have the potential barriers to accessing advice and treatment through community pharmacies been explored. In order to better understand some of the issues around the general health problems of methadone clients, whom they access for help and treatment, and barriers to utilising community pharmacists for help and treatment, a study was designed with the following aims:

• To review the general health problems that AMS clients managed within the specialist services suffered in a 3 month period;

• To document the incidence of chronic medical conditions;

• To explore who AMS clients turn to for advice;

• To explore whether clients utilise community pharmacists for this type of advice;

• To explore barriers to a greater role for community pharmacists.

## Methods

### Study design

The study employed a cross sectional design, using a self-completion questionnaire.

### Sample

All AMS clients managed within specialist services, but not those managed through authorised GP care, and who had appointments within the next three months were included in the study. AMS is not funded to provide general healthcare to methadone clients, thus those managed by GPs were not included because they already had access to healthcare via their GP-managed methadone treatment and thus may have biased the results.

### Sampling procedure

AMS clients managed through the CADS services were identified through the AMS database, and a list of AMS clients attending each CADS clinic on a "once every three months" basis was provided to the admissions officers (AOs) at each of the five CADS clinics. As a client presented for their appointment, he or she was asked whether they would be willing to complete a brief questionnaire either whilst waiting for their appointment or take it home and complete it later, returning it in a prepaid envelope provided for its return. Once a client had been approached the AO ticked off the client from the list, ensuring clients were only approached once.

This process took place between mid January and mid April 2005, thus capturing a 3-month time period in which, theoretically, each client would be seen by the service. In reality, a number of clients did not make or attend appointments, and the denominator for this study was therefore the number of clients attending appointments during this time period.

### Questionnaire design

A self-completion quantitative questionnaire was developed to explore perceived health status and treatment-seeking behaviour for individuals. Although other instruments are available that measure heath and wellbeing such as SF36 [26], the Quality of Wellbeing Scale [27] and Sickness Impact Profile Scale [28], they tend to focus on global health issues and are not designed with opiate users in mind. The health sections of the Opiate Treatment Index [29] contain a number of questions on health. However, the instrument is designed to explore health in a four-week period in a clinical setting, and is not designed for self-completion. In this study, the aim was to explore incidence of general health issues such as minor ailments which may or may not be specific to drug use, as a way of gauging potential for a community pharmacist role. From this perspective a three month time period was used, (four weeks being considered to short a period to capture the incidence of rarer health problems), and a selection of general health items was chosen that could be responded to by community pharmacists, and for which there are over-the-counter remedies available. Other more drug-specific items were included after discussion with the AMS clinical team, who wished to utilise the opportunity provided by the study to collect data on the clients. The questionnaire was reviewed by consumer advisors, AMS medical officers and case managers. Questionnaires were pre-piloted amongst staff and consumer advisors at CADS, and feedback indicated it was easy to complete, taking 5–10 minutes.

Questionnaires were anonymous – no client identifiers were required. Health problems surveyed include general health problems and symptoms suffered in the previous three months (e.g. cold, indigestion, nausea, diarrhoea, sweating, constipation, toothache, loss of appetite, abscesses, swollen hands and feet/fluid retention), psycho-neurological problems (headaches, difficulty sleeping, fatigue, depression), respiratory problems (hay fever cough, dry mouth), dry and itching eyes, and skin rashes. The existence of chronic health problems was also explored. Help- and treatment-seeking behaviour, barriers to help-seeking, and substance use were also surveyed.

Data were entered into SPSS version 12.1, and analysed using general frequencies. Further analysis was conducted using appropriate parametric and non-parametric statistical tests.

## Results

During the study period 715 appointments with AMS clients were scheduled, and a total of 556 clients (77.7%) were seen at appointments. Of these 556, 335 (60.3%) were male, 424 (76.3%) were European/Pakeha, 65 (11.7) Maori, 8 (1.4%) Pacific, 8 (1.4%) Asian and 51 (9.2%) other. Of these, 361 clients (65%) (57.9% male) were 'offered' a questionnaire, according to the records kept by AOs at each CADS clinic. Of these, 231 (64%) completed and returned a questionnaire (representing 42% who had appointments). Just over half (50.2%) were male (data missing on 14 cases), and the majority self-identified solely as European/Pakeha (72.7%). Maori (including those identifying as Maori and Pakeha) clients comprised 15.5%, Pacific Islanders 1.8%, Asian 0.9% and others 2.2% of the respondents (data missing on 16 cases). Just over half of the respondents (52.9%) stated they had a GP with whom they could comfortably discuss health problems (data missing on 23 cases).

Respondents had used the following substances once a week or more frequently in the previous three months: morphine 14.5%, cannabis 40.5%, stimulants (e.g. methamphetamine) 11.5%, ecstasy 2.2%, alcohol 23.3% and cigarettes 74.4% (data missing on 8 cases).

Table 1 provides data on self-reported chronic health problems. The most common condition that the respondents reported suffering from was hepatitis C (51.6%). About a quarter of the respondents (24.0%) reported mental health problems, 23.1% reported suffering from chronic pain, and 21.3% suffered from migraine. Only 2.3% of the respondents had diabetes and none of them reported being HIV positive.

**Table 1:** Self-reported medical conditions* (N = 221)

	**N**	**(%) **
Any self reported medical condition from list below	186	84.2
Hepatitis C	114	51.6
Mental health problems	53	24.0
Chronic pain	51	23.1
Migraine	47	21.3
Asthma	40	18.1
Hayfever or other allergies	36	16.3
Eczema/dermatitis	20	9.0
High blood pressure	18	8.1
Arthritis	12	5.4
Hepatitis B	12	5.4
Diabetes	5	2.3
HIV	0	0

*identified by ticking a box. No tick assumed to be not suffered from the condition

Clients were asked to identify health problems suffered in the last three months from a list provided (see Table 2 for details), by ticking a box for 'yes'. A blank box was assumed to be a negative response. The most common health problem experienced by these clients in the past three months was sweating (70.0%). More than half of the respondents also reported experiencing headache, fatigue and depression. The least frequently experienced conditions were hay fever (12.9%) and abscesses (12.1%). Respondents had suffered from a mean of 6.76 conditions (sd = 4.12; median = 6; mode = 4; range = 0–19).

**Table 2:** Health problems suffered in last 3 months* (N = 231)

	**N**	**%**
No health problems	3	1.3
Cold	56	24.2
Hayfever	28	12.1
Headaches	130	56.3
Indigestion	54	23.4
Cough	56	24.2
Nausea	80	34.6
Diarrhoea	39	16.9
Sweating	162	70.1
Skin rashes	49	21.2
Dry/itchy eye	36	15.6
Dry mouth	104	44.2
Constipation	105	45.5
Sleep problems	135	58.4
Toothache	108	46.8
Fatigue	117	50.6
Depression	117	50.6
Swollen hands/feet	66	28.6
Loss of appetite	90	30.9
Abscesses	32	13.9

* identified by ticking a box. No tick assumed to be not suffered from the problem

Clients were asked to tick up to three individuals or groups from a list provided, that they were most likely to ask for help when suffering from any health-related problems mentioned in Table 2, by ticking a box for 'yes'. A blank box was assumed to be a negative response. The most popular choices were the GP (56.7%), the client's partner (31.6%) and community pharmacists (27.9%). None of the respondents identified Maori health workers nor Hapu, Iwi health kaimahi among their first three choices. Five respondents stated that they would not seek help from anyone apart from themselves (see Table 3).

**Table 3:** Individuals or groups most likely to be consulted with regard to health problems in Table 2. (N = 215)

**Individual or group **	*Most likely to consult (can tick up to 3)		**Who else would consult (can tick as many as required)**	
	**N**	**%**	**N**	**%**
GP (doctor)	122	56.7	84	36.4
Partner	68	31.6	42	18.2
Pharmacist in chemist shop	60	27.9	95	41.1
Family/whanau^1 ^member	47	21.9	62	26.8
AMS Case manager	45	20.9	93	40.3
Friend	43	20.0	76	32.9
Chemist shop staff	15	7.0	37	16.0
Needle exchange staff	1	0.5	17	7.4
Maori health worker	0	0.0	5	2.2
Hapu, Iwi health kaimahi^2^	0	0.0	2	0.9
Other (includes Alcohol and drug hotline, CADS doctor, dentist, fellow methadone programme client, (mental health) nurse, therapist, naturopath, cleric).	12	5.6	11	4.8

*(NB respondents could tick up to three from a list. Those ticking more than three were excluded from this analysis).

^1^Whanau is a Maori word which means "the extended family which includes the nuclear family, and aunts, uncles and cousins".

^2^Hapu, Iwi health kaimahi – a community worker based in a local Maori community

Similarly, the respondents were then asked to identify who else they would consider asking for advice/help (see Table 3). Collating responses to 'who would you be most likely to consult' and 'who else would you consult', 93% of respondents indicated GPs, 69% community pharmacists and 61% AMS case managers. Around half indicated lay people such as family, friends or partner.

Respondents were asked to indicate, from a list provided, why they would not contact a community pharmacist for help, by ticking a box for 'yes'. A blank box was assumed to be a negative response (respondents could choose up to 3 reasons) – see Table 4. The most common reason for not seeking help from a community pharmacist was financial. The second and third most common reasons for not asking for help were that people preferred to wait for the problem to get better, and that they believed pharmacists did not understand their problems. In addition, 15.3% of people would not ask a pharmacist for help as they felt lack of privacy was an issue, and 12.4% of them had concerns about confidentiality.

**Table 4:** Reasons for AMS clients not seeking treatment or advice from a community pharmacist when suffering from health problems identified in Table 2 (N = 208)

	**N**	**%**
Cannot afford it	88	42.1
Prefer to wait until the problem gets better	77	36.8
Believing pharmacist lacks understanding about client health problems	38	18.2
Not private enough	32	15.3
Concerns about confidentiality	26	12.4
Prefer to seek treatment and advice from someone else	25	12.0
Time consuming	13	6.2
Have medicine at home	9	4.3

(NB respondents could tick up to three from a list. Those ticking more than three were excluded from this analysis).

(Data missing on 1 case)

Respondents were also asked to indicate how many times they had seen a GP, pharmacist, case manager, dentist, AMS doctor or needle exchange staff for help in the previous three months. Overall the number of consultations with health professionals was very low. Data were not normally distributed with some individuals being high users (e.g. 10–15 consultations in last three months), and the majority having no consultations. With the exception of GPs (median = 1), the median for all other health professionals was 0 (data missing on 14 cases).

## Discussion

This study represents the first published study which has specifically explored New Zealand methadone clients' primary healthcare needs and from whom they access treatment. The results indicate that this is a group that suffers a range of health problems similar to those encountered in the general population. Problems considered to be associated with drug use (and opiate use in particular) were reported by a large proportion of users – e.g. dental problems, insomnia, depression and constipation. Respondents also reported a high mean number of individual health problems, indicating there is a need for provision of primary healthcare to this group. Care in interpreting these results may however be needed for items such as depression, which are based on self-report and may not have actually been diagnosed.

The most likely individual to be contacted for support with healthcare was the GP, followed by a person's partner. Indeed friends and family also featured quite strongly, possibly indicating a high reliance on the lay health network over health professionals. Reasons for this need to be explored further, but results from this study indicate that for pharmacists at least, cost is a major barrier – being cited by two fifths of respondents and 'waiting until things get better' being second and a possibly proxy for cost. In the UK, the National Health Service has introduced a 'minor ailments scheme' in which community pharmacists are able to 'prescribe' to individuals in certain financially disadvantaged groups (though not specifically drug users) 'over-the-counter' (OTC) treatments free of charge [30,31]. Such a scheme might be considered for methadone clients. However, further study needs to be undertaken into the cost of non-treatment.

Other barriers to accessing help from pharmacists included lack of privacy and concerns about confidentiality. These results echo those found in studies of drug using clients in Scotland [23-25]. The issue of privacy may be addressed by the introduction of private areas and in Scotland funding has been provided to pharmacists to do this as an asset for all patients. The issue of confidentiality needs to be addressed through client education – the pharmacists' code of ethics specifically precludes breach of confidentiality except in issues where a duty of care may supersede a code of confidentiality. Nonetheless, pharmacy staff may need to be specifically educated with regard to this.

A number of respondents had concerns about whether community pharmacists were knowledgeable enough to provide such healthcare. In general with regard to minor ailments this should not be an issue. However, there are drug-related contextual issues that may need to be covered in tailored, professional development programmes such as the relationship between drug use and dental problems, managing pain with OTC products and when to refer and the need for this requires further exploration. Another approach to overcoming this barrier is to provide education for methadone clients on the role of the pharmacist and their ability to manage such health problems.

Respondents reported a high rate of use of other substances such as cannabis, tobacco and alcohol. With regard to smoking, American studies have shown that many methadone clients want to quit and are favourable towards the use of nicotine-replacement-therapies (NRT) [32]. Community pharmacists have been shown to be effective in helping smokers to quit [33] and with appropriate collaboration with drug services may have an important role to play.

A number of limitations should be taken into account in interpreting and extrapolating results from this study. The response rate of those who were 'offered' a questionnaire was 65%, which is high for a survey that was unable to employ a non-responder follow-up methodology. However, there are issues with regard to those who were not 'offered' a questionnaire – i.e. did they actually decline to take part, or were they simply missed during a busy clinic day. Furthermore, despite a requirement for clients to be seen once every 3 months, only 556/715 attended appointments. It maybe that those not attending are very stable on treatment; however, there may also be some who did not attend because they were unwell, and as such this potential bias needs to be taken into account. Thus, while a 65% response rate from those given a questionnaire was high, generalisations to the whole of the methadone clients in the study should be made with caution. The sample of respondents also under-represents males and slightly over-represents Maori when compared with data for all attendees during the study time period.

In designing the study it was initially envisaged that only those 'minor ailments' that might be attended to by community pharmacists would be included in the study. However, feedback from AMS staff indicated that they would like a number of other conditions or symptoms included such as depression, loss of appetite and abscesses – issues much less likely to be managed in community pharmacy. When asking respondents whom they would be most likely to contact about any of these health issues, and with high proportions reporting they suffered from depression for example, the results are thus likely to be skewed away from the community pharmacist. Ideally, respondents would have been asked whom they would have consulted for each condition, but this would have made the questionnaire too complex and may possibly have compromised a good response rate.

## Conclusion

Methadone clients in this study indicated that they suffered a number of general health problems, but in many cases were likely to seek help from a GP or their own partner, before seeking help from pharmacists. They also indicated that they might simply wait for problems to resolve. However, a significant proportion cited the community pharmacist as a person whom they would be most likely to contact, and the barriers towards consulting pharmacists in the main, are resolvable. In particular issues around cost, pharmacist training and reorientation of premises to allow for improved privacy need to be taken into consideration when attempting to expand a harm reduction role for pharmacists with this client group. Furthermore, there seems to be a need to address clients' beliefs about the community pharmacist's role in the provision of healthcare.

## Competing interests

The author(s) declare that they have no competing interests.

## Authors' contributions

JS designed the study and data collection instruments, supervised the researchers, analysed the data and drafted the manuscript. AW facilitated the process of data collection, supervised the researchers and contributed to the writing of the manuscript. CW helped with the design of the questionnaires, facilitated data collection and contributed to the writing of the manuscript. All authors read and approved the final manuscript.
